# Higher risks of hyperopia, myopia, astigmatism, and strabismus in children with autism spectrum disorder: a nationwide, population-based cohort study

**DOI:** 10.47626/1516-4446-2023-3515

**Published:** 2025-01-22

**Authors:** Yi-Lung Chen, Cheng-Fang Yen, Yu-Hung Lai, Ray C. Hsiao, Wei-Po Chou

**Affiliations:** 1 Department of Psychology, Asia University, Taichung, Taiwan; 2 Department of Healthcare Administration, Asia University, Taichung, Taiwan; 3 Department of Psychiatry, Kaohsiung Medical University Hospital, Kaohsiung, Taiwan; 4 Department of Psychiatry, School of Medicine, College of Medicine, Kaohsiung Medical University, Kaohsiung, Taiwan; 5 College of Professional Studies, National Pingtung University of Science and Technology, Pingtung, Taiwan; 6 Department of Ophthalmology, Kaohsiung Medical University Hospital, Kaohsiung Medical University, Kaohsiung, Taiwan; 7 Department of Ophthalmology, School of Medicine, College of Medicine, Kaohsiung Medical University, Kaohsiung, Taiwan; 8 Department of Psychiatry and Behavioral Sciences, University of Washington School of Medicine, Seattle, WA, USA; 9 Department of Psychiatry, Seattle Children’s, Seattle, WA, USA

**Keywords:** Autism spectrum disorder, hyperopia, myopia, astigmatism, strabismus

## Abstract

**Objective:**

In this population-based cohort study, we compared the risks of incident hyperopia, myopia, astigmatism, and strabismus between children with autism spectrum disorder (ASD) and those without ASD.

**Methods:**

This study included children who were born in Taiwan at any time between 2004 and 2017, using data from the Taiwan Maternal and Child Health Database (TMCHD). We included 20,688 children with ASD and 2,062,120 matched controls to estimate the risks of incident hyperopia, myopia, astigmatism, and strabismus. Cox proportional hazards regression models were conducted for risk assessment. Models were adjusted for sex, calendar year of birth, and gestational age at birth. Statistical significance was determined through adjusted hazard ratios (aHRs) and 95%CIs.

**Results:**

Children with ASD had higher risks of incident hyperopia (aHR: 1.78; 95%CI 1.70-1.86), myopia (aHR: 1.27; 95%CI 1.24-1.30), astigmatism (aHR: 1.51; 95%CI 1.46-1.56), and strabismus (aHR: 2.18; 95%CI 2.05-2.32) compared to children without ASD.

**Conclusion:**

Clinicians should screen children with ASD for potential eye conditions. Further studies are required to elucidate the mechanisms linking ASD with eye diseases. In addition, studies should explore how the type and severity of ASD symptoms influence the detection of these eye conditions.

## Introduction

Several studies have investigated visual function and perception in patients with autism spectrum disorder (ASD).^1^,^2^ However, researchers have debated on whether children with ASD undergo normal visual development^3^,^4^ or experience ophthalmic abnormalities such as refractive errors and strabismus.^5^-^9^ In a retrospective chart review, Chang et al.^10^ reported that 71% in children with ASD had eye conditions, with refractive errors (42%) and strabismus (32%) being the most common. Similarly, Lau et al.^11^ found that 62% of children with ASD had refractive errors and 63% had strabismus. Other retrospective studies, including those by Kabatas^6^ and Black,^7^ reported prevalence rates of eye conditions in patients with ASD at 27% and 52%, respectively. A cross-sectional study conducted by Milne^4^ indicated that many aspects of vision, including visual acuity, remain unaffected in patients with ASD. However, a cross-sectional study found that more impairment in visual motor such as convergence insufficiency, stereopsis deficit, and hyperopia in children with ASD.^12^ The inconsistent findings across these studies may be due to variations in sample size, age groups, or retrospective study designs. To address these limitations, this nationwide, population-based cohort study aims to examine the associations between four common pediatric eye conditions – hyperopia, myopia, astigmatism, and strabismus – and ASD in children. We hypothesized that the risks of developing these conditions would differ between children with ASD and those without ASD.

## Methods

### Data source

We obtained data from the Taiwan Maternal and Child Health Database (TMCHD), a longitudinal dataset from the National Health Insurance Research Database (NHIRD). The TMCHD includes data on approximately 99.78% of all children born in Taiwan between 2004 and 2017.^13^ It provides detailed information such as birth data (e.g., sex, birth weight, and birth date), medical history, and diagnosis codes from the International Classification of Diseases, Clinical Modification (ICD-9-CM).^2^

### Study population, exposure, outcomes, and covariates

Diagnoses were identified by using diagnostic codes from the ICD-9-CM and ICD-10. The relevant codes for the five conditions studied were as follows: ASD – ICD-9-CM code 299 and ICD-10 code F84; hyperopia – ICD-9-CM code 367.0 and ICD-10 code H52.0; myopia – ICD-9-CM codes 360.21 and 367.1 and ICD-10 codes H44.2 and H52.1; astigmatism – ICD-9-CM codes 367.20-367.22 and ICD-10 code H52.2; and strabismus -ICD-9-CM codes 277.8, 378.50-378.53, and 378.00-378.13 and ICD-10 codes H49.00-H49.4, H49.8, H49.9, H50.0-H50.6, H50.8, and H50.9. ASD diagnoses were made by board-certified psychiatrists based on clinical interviews with the children and their primary caregivers. Hyperopia, myopia, astigmatism, and strabismus were diagnosed by board-certified ophthalmologists using clinical judgment, medical history, and instrument-based inspections.

The case group consisted of children who had received at least two outpatient diagnoses or one inpatient diagnosis of ASD. The control group included children aged 4 years or older to allow enough time for symptom observation and accurate ASD diagnosis. The date of birth was used as the index date for calculations.

The primary outcomes were new-onset hyperopia, myopia, astigmatism, and strabismus. To identify incident ophthalmic conditions, we excluded individuals with a prior history of these conditions in the ASD group. This exclusion was not necessary for the non-ASD group, as they did not have an ASD diagnosis. For the ASD group, follow-up began on the date of ASD diagnosis and continued until either the diagnosis of an ophthalmic condition, the date of death, or the end of the study (December 31, 2018). For the non-ASD group, follow-up began at birth, as they had no ASD diagnosis.

Children were followed from their birth date until the date of diagnosis, the date of death, or the end of the study period (December 31, 2018). We adjusted for the effects of several covariates, including the calendar year of birth, sex, gestational age at birth as well as the presence of cerebral palsy (ICD-9-CM code 343, ICD-10 code G80) and seizures (ICD-9-CM code 345, ICD-10 codes G40-G41).

### Statistical analyses

All statistical analyses were performed using SAS version 9.4. Categorical variables are presented as frequencies and percentages, while continuous variables are expressed as means and SD. Between-group differences were evaluated using Pearson’s chi-square test for categorical variables and independent *t* tests for continuous variables. To assess associations between study variables, Cox proportional hazards regression models were constructed, using time-on-study as the time scale. The models were adjusted for sex, calendar year of birth, and gestational age.^14^ Adjusted hazard ratios (aHRs) with 95% confidence intervals (CIs) were calculated to validate longitudinal associations. Kaplan-Meier survival curves were generated for time-to-event variables, and a log-rank test was used to compare the survival curves. Schoenfeld residuals were computed to verify the assumption of proportional hazards between groups.

### Ethics statement

This study was approved by the Research Ethics Committee at China Medical University Hospital (approval code: CMUH108-REC1-142).

## Results

This study included 20,688 children with ASD (17,153 boys and 3,535 girls) and 2,062,120 children without ASD (1,067,988 boys and 994,132 girls). The demographic characteristics of the study cohort are summarized in Table 1. The proportion of boys was significantly higher in the ASD group compared to the control group (82.9% vs. 51.8%, respectively; p < 0.001). Incidence rates of three eye conditions were notably higher in the ASD group compared to the control group: hyperopia (3.7% vs. 1.8%, p < 0.001), astigmatism (9.7% vs. 6.9%, p < 0.001), and strabismus (3.1% vs. 0.8%, p < 0.001). However, there was no significant difference in the incidence of myopia between the two groups (21% vs. 21.8%, p > 0.05).

**Figure 1:**
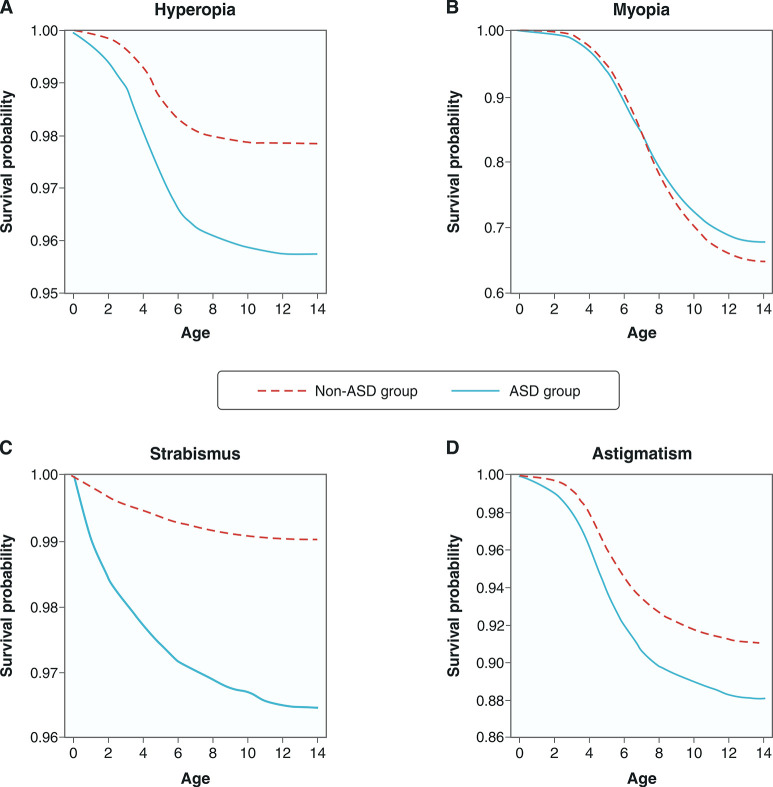
Kaplan-Meier curves for eye condition-free survival in children with and without ASD. ASD = autism spectrum disorder.

Table 2 shows that after adjustment for covariates, the ASD group had a significantly higher risk of developing hyperopia (aHR: 1.78; 95% CI: 1.70-1.86), myopia (aHR: 1.27; 95% CI: 1.24-1.30), astigmatism (aHR: 1.51; 95% CI: 1.46-1.56), and strabismus (aHR: 2.18; 95% CI: 2.05-2.32) compared to the control group. Kaplan-Meier survival curves demonstrated the time from enrollment to the occurrence of hyperopia, astigmatism, and strabismus was shorter in the ASD group than in the control group (Figures 1A-D).

## Discussion

This is the first large-scale, nationwide, register-based cohort linking eye conditions with ASD. It suggests that children and adolescents with ASD have at higher risks of incident hyperopia, myopia, astigmatism, and strabismus than did those without ASD. By addressing previous mixed results on this association, our study provides stronger evidence of the increased risk of eye conditions in children and adolescents with ASD, using a robust methodology and a statistically significant sample size. Our findings underscore the importance of early detection of ophthalmic comorbidities in children with ASD. To reduce the prevalence of vision problems in this population, healthcare professionals should receive specialized training to better identify visual impairments in these children.^15^ Children with ASD should be screened for signs of visual impairment as early as possible.

Since the first study by Keeler,^16^ which described autistic behaviors in five totally blind preschool children, several epidemiological studies have examined the relationship between ASD and eye conditions. These studies have reported rates of eye conditions in children with ASD ranging from 30% to 70%.^6^,^7^,^10^ However, most previous research on this topic has been based on retrospective chart reviews or cross-sectional studies, which could not establish a clear temporal relationship between ASD and eye conditions.^7^,^10^,^11^,^14^ In contrast, our study utilized longitudinal data to better clarify the timing of these associations.

Children with eye conditions face challenges in learning, social interaction, and communication.^17^ For example, children with visual impairment exhibit clinical symptoms that resemble autistic behaviors, such as repetitive eye-poking and body rocking.^18^ They are also more likely to experience social isolation and engage in solitary play compared to their peers without eye conditions.^17^,^18^ However, whether these sensory abnormalities are a cause or consequence of abnormal social behaviors remains debated.^17^,^19^ Other factors may contribute to both ASD and eye conditions. The associations between ASD and eye diseases may be mediated by shared neurological dysfunctions and inheritance. Both ASD and ophthalmic conditions share several neurological risk factors, including prematurity,^20^ seizure,^21^,^22^ low gestational age,^23^ and cerebral palsy.^24^,^25^

Genetic studies have shown that gamma-aminobutyric acid (GABA)-mediated neural transmission is disrupted in both patients with ASD^26^-^28^ and those with binocular visual disorders such as strabismus.^29^ In part, sensory atypicalities in ASD may result from differences in GABA receptor function.^26^ Studies have implicated the genes *FMR1* and *CSDE1* in ASD.^27^,^28^ The *FMR1* premutation is associated with primary visual cortex dysfunction,^27^ while *CSDE1* is associated with retinal development in humans.^28^ At the neurobiological level, the altered regulation of the excitatory-inhibitory balance, particularly in the inhibitory GABA pathways, may contribute to the changes in visual sensory processing in patients with ASD.^29^ Genetic factors involved in the dysfunction of the GABAergic system may partially mediate the associations between ASD and eye conditions, although further studies are needed to identify these genetic influences.^10^

This study has several strengths. First, it is a population-based cohort study with a large sample size. Second, the diagnoses were made by specialists, ensuring reliability. Lastly, the statistical models were adjusted for sex, calendar year of birth, and gestational age at birth, reducing bias from potential confounders. However, there are some limitations. First, it is unclear whether these findings are generalizable to Taiwanese children not receiving treatment for ASD or ophthalmic conditions, or to non-Taiwanese populations. Second, some ophthalmic diseases might remain undetected in children with ASD due to communication difficulties, potentially leading to an underestimation of disease prevalence. Third, the severity of ASD symptoms was not recorded in the TMCHD, preventing analysis of their impact on vision problems. Finally, we could not examine the effects of medications. Further studies are needed to determine whether treatments for ASD or vision correction influence the relationship between ASD and ophthalmic diseases.

In conclusion, this study suggests that children with ASD are at higher risk for hyperopia, myopia, astigmatism, and strabismus in a nationwide population cohort. This highlights the importance of early ophthalmological screening and treatment. Future research is needed to clarify the clinical mechanisms underlying these associations and to identify additional risk factors.

## Disclosure

The authors report no conflicts of interest.

## Figures and Tables

**Table 1:** Demographic characteristics, gestational age, and common eye diseases in children with ASD and children without ASD

Variable	ASD (n=20,688)	Non-ASD (n=2,062,120)	p-value
Age (years), mean (SD)	9.0 (3.2)	9.0 (3.1)	< 0.1(%)
Gestational age (weeks), mean (SD)	38.1 (2.1)	38.3 (1.7)	< 0.1(%)
Sex			< 0.1(%)
Boys	17,153 (82.9)	1,067,988 (51.8)	< 0.1(%)
Girls	3,535 (17.1)	994,132 (48.2)	< 0.1(%)
Covariate disease			
Seizures	279 (1.35)	155896 (7.56)	< 0.1(%)
Cerebral palsy	68 (0.33)	64544 (3.13)	< 0.1(%)
Eye conditions			
Hyperopia	769 (3.7)	37900 (1.8)	< 0.1(%)
Myopia	4,350 (21.0)	449,747 (21.8)	> 5(%)
Astigmatism	2,004 (9.7)	142,100 (6.9)	< 0.1(%)
Strabismus	646 (3.1)	17,071 (0.8)	< 0.1(%)
Any	5,948 (28.8)	530,329 (25.7)	< 0.1(%)

Data presented as n (%), unless otherwise specified.

ASD = autism spectrum disorder.

**Table 2:** Associations between ASD and common eye conditions in children

Model	aHR
Hyperopia	1.78 (1.70-1.86)
Myopia	1.27 (1.24-1.30)
Astigmatism	1.51 (1.46-1.56)
Strabismus	2.18 (2.05-2.32)

Adjusted hazard ratio was calculated after adjusting the statistical model for sex, calendar year of birth, seizures, cerebral palsy, and gestational age.

aHR = adjusted hazard ratio; ASD = autism spectrum disorder.
